# Use of Translational, Genetically Modified Porcine Models to Ultimately Improve Intestinal Disease Treatment

**DOI:** 10.3389/fvets.2022.878952

**Published:** 2022-05-20

**Authors:** Cecilia R. Schaaf, Liara M. Gonzalez

**Affiliations:** Department of Clinical Sciences, College of Veterinary Medicine, North Carolina State University, Raleigh, NC, United States

**Keywords:** cystic fibrosis, colorectal cancer, ischemia-reperfusion injury, genetically altered models, intestinal disease, translational porcine model, stem cell reporter model

## Abstract

For both human and veterinary patients, non-infectious intestinal disease is a major cause of morbidity and mortality. To improve treatment of intestinal disease, large animal models are increasingly recognized as critical tools to translate the basic science discoveries made in rodent models into clinical application. Large animal intestinal models, particularly porcine, more closely resemble human anatomy, physiology, and disease pathogenesis; these features make them critical to the pre-clinical study of intestinal disease treatments. Previously, large animal model use has been somewhat precluded by the lack of genetically altered large animals to mechanistically investigate non-infectious intestinal diseases such as colorectal cancer, cystic fibrosis, and ischemia-reperfusion injury. However, recent advances and increased availability of gene editing technologies has led to both novel use of large animal models in clinically relevant intestinal disease research and improved testing of potential therapeutics for these diseases.

## Introduction

Gastrointestinal disease accounts for over 3.0 million hospitalizations and over '135.9 billion in health care expenditures per year (1). To lessen this incredible burden to patients and our healthcare system, animal models play a critical role in discovery of intestinal disease pathogenesis and therapeutic innovation. For successful clinical translation, it is critical that animal models are properly validated. The criteria used to validate an animal model include certifying similarity in biology and clinical presentation between model and human disease (face validity), confirming that clinical interventions produce similar effects (predictive ability), and demonstrating that the target under investigation has a similar role in the model compared to human clinical disease (target validity) (2). Of intestinal disease animal models, rodents are historically preferred for use due to their low cost and maintenance, rapid reproduction, and readily available rodent-specific reagents. However, it is now widely recognized that rodents do not fully mimic human disease, physiology, immunology, or drug metabolism, thus limiting their use as pre-clinical models for disease treatment (3–7). For example, despite promising pre-clinical murine anti-cancer therapeutic studies, success rate of these therapeutics in human clinical trial is only around 5% (4, 8). Furthermore, the small size of rodents makes it difficult to model and advance surgical and endoscopic techniques. The differences between rodents and humans have left gaps in both basic science research and pre-clinical model development for intestinal diseases.

To better represent both human physiology and disease, and aid in the discovery of new treatments, porcine models are gaining popularity. With similar genome, size and architecture of the intestine, omnivorous diet, microbiome, immunology, and physiology to humans, pigs are increasingly the preferred model of enteric diseases (Table 1) (3, 6, 13, 17–23). The large size of the pig allows for multiple, longitudinal sampling from the same individual. Their large litter size of around 12 piglets allows for ease of gender and sibling matching. These attributes reduce both experimental variation as well as overall animal use. Additionally, for toxicology and drug discovery testing, pigs have oral and parenteral dosing rates similar to humans as well as similar responses to a variety of drug classes (24).

**Table 1:** Summary of comparisons between human and porcine intestinal physiology and anatomy as well as advantages and disadvantages of porcine models.

**Porcine intestine similarities to human**	**Porcine intestine differences from human**
• Intestinal length • Omnivorous diet • Microbiome (9) • Immune response resembles human in 80% of analyzed parameters (10) • High genome homology (11, 12)	• Presence of spiral colon • Inverted lymph node structure • Distribution and frequency of intestinal lymphocyte populations • Continuous ileal Peyer's patch
**Porcine model advantages**	**Porcine model disadvantages**
• Husbandry well understood • Outbred breeds better mimic variation between human individuals (13) • Large litters for gender/sibling matching • Large animal size allows for improving surgical/endoscopy techniques using human equipment for diseases like Cystic Fibrosis and colorectal cancer • Longer lifespan permits longitudinal studies • Oral/parenteral dosing and responses to many drug classes similar to humans (14) • More accepted on ethical basis compared to non-human primates or other large animals • Numerous *in vitro* applications such as advanced 3D organoid cultures (15, 16)	• Necessitate large, specialized housing facilities • More expensive than mice • Not as many species-specific reagents as mice

**Figure 1:**
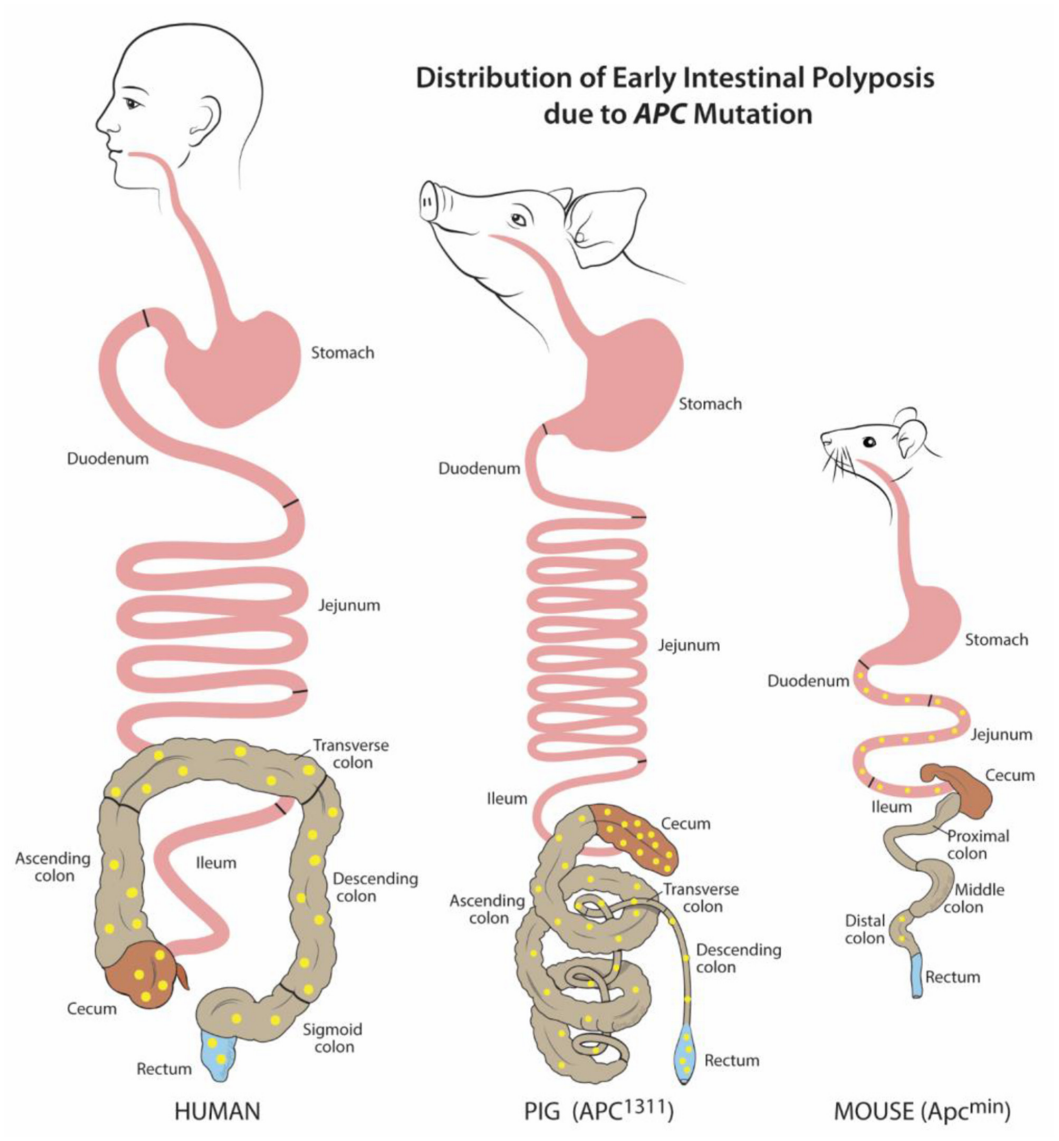
Distribution of early intestinal polyposis due to *APC* mutation. Human and porcine (*APC*^1311^) early adenoma/polyp distribution is similar within the colon and rectum, while murine (Apc^min^) is mostly localized to the small intestine. Adenoma/polyps shown in yellow. Figure adapted from Gonzalez et al. and Flisikowska et al. (21, 26).

Grossly, both human and porcine adult intestine are at a ratio of 0.1 length per kilogram of body weight (25). The anatomy of the small intestine is similar between pig and human, though the large intestine varies slightly in the pig due to a larger cecum, lack of appendix, and the presence of the spiral colon (Figure 1) (3). Microscopically, for both species, the small and large intestine are comprised of a single layer of epithelial cells, interspersed with intra-epithelial lymphocytes, to serve as a barrier between luminal contents and systemic circulation. The single layer epithelium covers villus projections (present only in small intestine) and extends down into the crypts of Lieberkuhn. Located at the crypt-base are the intestinal epithelial stem cells (ISCs), which are responsible for renewing the epithelial cell populations on a continuous 3–5 day cycle (17, 27–29). In the human, the ISCs are interspersed with Paneth cells, a specialized secretory cell type that both supports ISC function and releases antimicrobial factors into the intestinal lumen (30, 31). While a similar cell population expressing the same biomarkers and intracellular structures has been identified in the pig, the porcine Paneth cell has yet to be fully defined (15, 32).

Beneath the epithelial barrier is the lamina propria compartment. While the lamina propria is made up of a mix of structural elements including blood and lymph vessels, connective tissue, and mesenchymal cells, immune cells constitute a major population, including dendritic cells and lymphocytes. These immune cells are responsible for discriminating between harmless luminal antigens and potential enteropathogens (33–35). Some differences exist between the immunologic organization between human and pig intestine such as the distribution and frequency of lamina propria and intraepithelial lymphocyte populations, the inverted structure of porcine peripheral and gut-associated lymph nodes, and the aggregated lymphoid follicles (Peyer's patches) which form one long continuous band in the porcine ileum (22, 29, 35). However, despite these differences, pigs are still recognized as useful models in several enteric immunologic studies including infectious disease, oral vaccination, small bowel transplantation, food hypersensitivity, and immune development (13, 22, 36–39).

With these similarities in both physiology and architecture between human and porcine intestine (Table 1), porcine models of various intestinal injuries such as ischemia-reperfusion injury, intestinal transplantation, short gut syndrome, and necrotizing enterocolitis have progressed the field of gastroenterology (37, 40–44). Furthermore, *in vitro* advancements continue to broaden the utility of porcine enteric disease models. These advancements include an increase in porcine specific reagents and the use of primary intestinal epithelial cell culture in 2-D monolayers, 3-D organoid culture, and co-culture with microbes and lamina-propria derived cells to better understand intestinal barrier function (15, 16, 19, 20, 40, 41, 45). However, for more mechanistic studies and to better understand human genetic diseases in the wide array of intestinal maladies, advancements in porcine gene-edited models are needed. Fortunately, enhanced strategies to edit the porcine genome and develop transgenic models, as well as approaches to genetically modify porcine-derived intestinal organoids, have increased the availability of pre-clinical modeling for Cystic Fibrosis (CF), colorectal cancer (CRC), and ischemia-reperfusion injury (Table 2). This review summarizes the current use of pre-clinical, gene edited porcine intestinal disease and injury models and evaluates future additional needs to ultimately improve treatment of intestinal disease.

**Table 2:** Summary of findings from gene-edited porcine models of non-infectious intestinal disease.

**Gene-edited porcine model**	**Key findings for intestinal disease**	**Sources**
**Cystic fibrosis**		
*CFTR^−/−^*	Intestinal gene editing to correct cystic fibrosis transmembrane conductance regulator (CFTR) expression alleviates Cystic Fibrosis induced obstructions	(46)
**Colorectal cancer**		
*APC^1311^*	Adenomatous polyposis coli (*APC*) mutation model reproduces colonic and rectal polyps as seen in familial adenomatous polyposis (FAP)	(26)
	Identification of gene expression, micro-RNAs associated with FAP	(47–50)
	Development of nanoparticles to improve endoscopic identification of dysplastic lesions and adenomas	(51, 52)
*TP53^*R*167*H*^*	Porcine TP53 isoforms expressed similarly to humans; TP53 variants and circular RNA overexpressed in colon	(53, 54)
*KRAS^*G*12*D*^ TP53^*R*167*H*^*	Cre-recombinase inducible *TP53* and Kirsten rat sarcoma viral oncogene homolog (*KRAS*) mutation model leads to intestinal carcinoma development	(55)
**Ischemia-reperfusion injury**		
Enteroid culture	*In vitro* gene-editing of ischemic injured intestinal epithelium identifies cellular mechanisms of repair	(41)
**Future directions: stem cell reporter**		
LGR5-H2B-GFP	*In vivo* and *in vitro* tracking of LGR5^+^ intestinal stem cells, further model validation necessary	(56)

## Gene Therapy in Porcine Cystic Fibrosis Models to Alleviate Intestinal Obstruction

Cystic Fibrosis (CF) is a life-threatening disease due to various mutations in the CF transmembrane conductance regulator (*CFTR*) gene (57). This critical gene encodes for an anion channel widely expressed in epithelium including lung, pancreas, kidney, and intestine; its loss of function inhibits chloride and bicarbonate transport across cell membranes. This leads to thick mucoid secretions with low pH, subsequent pathogen colonization, and dysregulated inflammation (57, 58). While cause of death in patients afflicted with CF is primarily due to respiratory failure, intestinal disease also contributes significantly to patient morbidity (59). Up to 20% of infants with CF suffer from meconium ileus at birth, followed by distal intestinal obstructive syndrome due to intestinal atresia, diverticulosis, and microcolon (46, 57). While genetically modified murine models of CF assisted in basic understanding of *CFTR* functions, these mice fail to fully recreate human clinical disease (59). These limitations in the mouse models impeded further discovery of disease pathogenesis and potential therapeutics.

In 2008, in an effort to develop an animal model that better represents CF clinical disease, Rogers et al. utilized recombinant adeno-associated virus (rAAV) vectors to create the first *CFTR*-null piglets (*CFTR*^−/−^). These piglets also demonstrated immediate clinical signs of intestinal obstruction similar to that in human infants with CF (60). These clinical signs include intestinal atresia, microcolon, and diverticulosis (57). Other gene editing strategies have since been used to generate *CFTR*^−/−^ pigs including bacterial artificial chromosome vectors (61) as well as rAAVs to introduce a point mutation within the *CFTR* gene, *CFTR*-ΔF508 mutation (59, 62). This specific *CFTR* mutation is the most common CF-causing mutation in human patients – it accounts for about 70% of CF alleles (62). Murine models with this same induced point mutation fail to develop airway disease, pushing the need for a porcine model of this common mutation. While *CFTR*-ΔF508 porcine models display the same features of human disease in the lung and intestine, the rate of meconium ileus is 100% in the newborn pigs, in contrast to a rate of 20% in human infants (46). As in humans, meconium ileus requires immediate medical and/or surgical correction, which inhibited the use of the porcine model due to cost and complexity. These factors pushed researchers to utilize additional gene editing techniques to alleviate meconium ileus in the porcine model. Stoltz et al. were able to correct this phenotype by inducing *CFTR* expression under the control of intestinal fatty acid-binding protein (*iFABP*) in *CFTR*^−/−^ pig fibroblasts (46). These findings indicated that correcting the expression of *CFTR* by gene editing in the intestine is sufficient to prevent intestinal obstruction. Further work in the porcine CF models is necessary to identify exactly how much *CFTR* function is required for proper intestinal function. With these findings, novel gene therapy approaches can be developed such as somatic tissue gene editing to restore endogenous *CFTR* function. It is critical that this research is done in a porcine model, similarly sized to humans, so that delivery of therapeutics can be modeled using human equipment such as endoscopy. The information gained from a translational porcine model of CF will lead to the development of innovative gene therapy treatment approaches to alleviate CF-induced intestinal obstruction.

## Improved Modeling and Detection of Colorectal Cancer in Gene Edited Pigs

As of 2020 in the US, colorectal cancer (CRC) is the second leading cause of cancer related deaths for men and women combined (63). For many patients, including those with precursor familial adenomatous polyposis condition (FAP), CRC begins with germline or somatic mutations in the tumor-suppressor, adenomatous polyposis coli (*APC*) gene. This key driver mutation initiates polyp formation in the colonic epithelium. Subsequent compounding epigenetic changes and genetic mutations progresses tumorigenesis through the adenoma-carcinoma sequence, often culminating in metastatic cancer (64). For reasons unknown, CRC incidence has recently risen for young and middle-aged adults. Furthermore, many CRC tumor subtypes exist which remain without treatments (63). Without a doubt, there is a critical need for animal models to better understand CRC pathogenesis and develop targeted therapies. Attempts to recreate the polyp-adenoma-carcinoma pathogenesis sequence by mutating *Apc* (*Apc*^+/min^) in the mouse usually only leads to non-invasive, non-metastatic neoplasia. Furthermore, this neoplasia only typically occurs in the murine small intestine instead of the colon (Figure 1) (26, 65–67). Moreover, the small size of mice and critical differences in drug metabolism make these mice impractical for development of human CRC drug therapies, progress in endoscopic imaging techniques, or improving surgical interventions.

To overcome these barriers and create an improved, human-scale CRC model, the first gene-targeted APC mutated pig line was developed by inserting a translational stop signal at codon 1311 (*APC*^1311^*)*. This mutation is orthologous to the germline mutations in patients with familial adenomatous polyposis condition (FAP) (26). In these genetically modified pigs, colonic, and rectal polyps and adenomas develop such as those found in human FAP and CRC patients (Figure 1) (68). Since their development, these pigs have contributed greatly to the discovery of epigenetic modifications, dysplastic polyp premalignant progression, and the function of genes other than *APC* that contribute to the varying severity and progression of disease in patients with FAP (47–49, 69). One pivotal study by Stachowiak et al., using *APC*^1311^ pigs, was the first to reveal that microRNAs are associated with premalignant transformation of colon polyps and can serve as potential useful biomarkers of disease development (50). Tan et al. attempted to replicate the *APC* mutation porcine model by transcription activator-like effector nucleases (TALENs) introduction of a stop signal at codon 902; however, these pigs have yet to develop CRC phenotype (70).

In addition to the *APC*^1311^ line, other mutated tumor suppressor gene porcine models are now available to study tumorigenesis. One such group carries a latent Cre-activated tumor protein *p53* gene (*TP53*) mutated allele (*TP53*^*R*167*H*^) (53). This mutation is orthologous to the oncogenic human mutant *TP53* allele that plays a role in numerous human cancers including CRC. Previously, studies using the *APC*^1311^ pigs reported that in severe cases of polyposis, there is an increase in expression of polymorphic *TP53* (49). A more recent study using the *TP53*^*R*167*H*^ pig demonstrated that these pigs express *TP53* isoforms in a more similar manner to humans, further underscoring the benefits of porcine cancer models compared to murine. The *TP53*^*R*167*H*^ porcine model showed that *TP53* variants and circular RNA are overexpressed in the colon, indicating likely oncogenic function (54). These findings highlight the important role of porcine oncogenic models to improve our understanding of the genetic and epigenetic changes that contribute to CRC pathogenesis. Further developments on the *TP53*^*R*167*H*^ model include the addition of inducible Kirsten rat sarcoma viral oncogene homolog (*KRAS*) mutation (55, 71). Mutated *KRAS* is present in over 25% of human tumors, including CRC, and is one of the more commonly activated oncogenes (72). Schook et al. showed that these Cre recombinase inducible *KRAS*^*G*12*D*^ *TP53*^*R*167*H*^ transgenic pigs developed rapid and reproducible mesenchymal tumors. For more specific study of intestinal cancer, Callesen et al. refined the combined *KRAS* and *TP53* mutation model by directing the control of the inducible recombination events under an intestinal epithelial specific gene promoter, which led to development of duodenal carcinoma. Further model establishment studies using the *KRAS*^*G*12*D*^ *TP53*^*R*167*H*^ pigs are warranted to establish carcinoma development in the lower intestine in order to better understand CRC carcinogenesis in a translational large animal model.

While these gene-targeted porcine CRC models have served an important role for cellular mechanistic studies, they can also afford clinicians and researchers opportunities to improve endoscopy skills for minimally invasive surgery, conduct longitudinal sampling and monitoring during treatment, and enhance detection systems for pre-malignancies in a human-sized animal model. In general, porcine models have been popular for testing and advancing endoscopic techniques, particularly for colonoscopy (73–75). Early diagnostic detection of colon dysplasia and adenomas typically relies on white-light endoscopy (76). However, due to the subtle appearance of adenomas *in situ*, early-stage CRC lesions are often missed, especially in patients with abnormal colonic mucosa due to inflammatory bowel disease (51, 52). To improve real-time detection of colorectal adenomas, one group developed biodegradable near-infrared fluorescent silica nanoparticles (FSNs) (52). These FSNs, administered intravascularly, permeate into cancerous tissue and ‘mark’ a lesion because tumor and dysplasia-associated blood and lymph vessels are typically leaky (52, 77). Additionally, development of these biodegradable FSNs ensured no long-term sequestration within the body, as is typical of traditional nanoparticles (52). In that study, nanoparticle application was tested by administering FSNs to *APC*^1311^ pigs intravenously. Twenty-four hours later, colons were surveyed using near-infrared fluorescence-assisted white light endoscopy and adenomas were successfully highlighted by the FSNs (52). Since this study, additional work has successfully tested other probes in the *APC*^1311^ pigs to serve as CRC polyp markers (51). This pre-clinical application testing made possible by the *APC*^1311^ pigs was critical to develop new techniques to accurately identify clinically significant colorectal dysplasia in human patients.

## Gene Editing in Porcine *In vitro* Models of Ischemia-Reperfusion Injury

Ischemic injury occurs when there is reduction or complete loss of blood flow to an organ. In the intestine, ischemic events can occur due to numerous pathologic events including thrombi, emboli, shock, cardiac insult, mechanical obstruction such as a hernia or intussusception, or necrotizing enterocolitis (21). In all of these disease states, the decrease in blood flow to the intestine diminishes the oxygen supply necessary for normal cellular metabolism. Cell damage and apoptosis quickly follows, particularly within the intestinal epithelium that is responsible for maintaining a critical barrier between harmful luminal microbes and systemic vasculature. Microbial translocation across a compromised epithelial barrier can develop into systemic inflammatory response syndrome, intestinal necrosis, and remote organ failure. Ultimately, this disease progression results in over 50% patient mortality (78). To lessen the high mortality rate, intestinal ischemia animal models are critical to better understand the pathophysiology of ischemic injury, identify factors driving epithelial repair, and develop potential therapeutics (40–42, 79).

The process of ischemia-induced epithelial cell loss, as described in numerous animal models, begins at the villus tip and progressively extends down to the crypt-base intestinal epithelial stem cell (ISC) compartment with increasing durations of ischemia (40, 80–83). In the ISC compartment, two populations of ISCs exist: active, proliferating ISCs that are sensitive to injury (aISCs) and quiescent, reserve ISCs that are injury resistant (rISCs). These two populations were first described using murine models (84, 85). However, small rodent models are unable to accurately represent severe human ischemic injury, likely due to differences in intestinal microvascular anatomy and overall small intestinal size (17). Thus, the impact of ischemic injury on these two ISC populations was largely undescribed until the introduction of a porcine surgical model of mesenteric vascular occlusion (16, 17). With similar sized intestine and more similar microvascular anatomy as humans, pigs make for a better model of intestinal ischemia (17, 86). Using the surgical porcine model of mesenteric vascular occlusion, researchers identified that severe ischemic injury differentially impacts the two known ISC populations: aISCs undergo apoptosis while rISCs are preserved and are likely responsible for epithelial recovery after injury (40). In this model, rISCs were identified *in vivo* by expression of the known ISC biomarker homeodomain only protein X (HOPX) (85). When ischemic-injured tissue, initially enriched in HOPX^+^ rISCs, was recovered *in vivo* for up to 3 days post injury, increased signs of crypt-base epithelial regeneration corresponded to a decrease in HOPX expression (41). Until this point, HOPX, a known tumor-suppressor gene in other cell types, had served as merely a biomarker of rISCs (87). To clarify the potential role of HOPX as a controller of ISC proliferation after severe ischemia, genetic modification of the porcine ischemic injury model was necessary.

Whole animal, genetically-modified porcine models have yet to be used in intestinal ischemia-reperfusion studies. However, recent advances in genetic modification of porcine intestinal crypt culture are a promising first step for more mechanistic studies. Culture techniques for porcine enteroids, 3D organoids derived from intestinal stem cells that recapitulate the intestinal epithelium, have been well described (15, 16, 28). Using lentivirus, Khalil et al. were the first group to genetically modify uninjured porcine intestinal crypts to create GFP expressing enteroids (88). To better understand epithelial recovery after ischemic injury and the specific role of HOPX function in epithelial crypt cells, Stewart et al. utilized adenovirus mediated transduction of short hairpin RNA to silence *HOPX* within ischemic injured crypt epithelium and showed that HOPX serves to suppress cellular proliferation in resultant enteroids (41). This novel advancement in porcine enteroids encourages future experiments of *in vitro* gene editing to improve our understanding of repair mechanisms in clinically relevant intestinal ischemia.

## Future Directions: Development of Transgenic Porcine Intestinal Stem Cell Reporter Models

To improve studies of the cellular mechanisms involved in various types of intestinal injury and cancer, transgenic reporter porcine models are warranted for *in vivo* and *in vitro* cell tracking of intestinal cell populations. Intestinal epithelial stem cell reporter pigs are of particular interest as ISCs play critical roles in both the generation of colorectal cancer and in epithelial barrier regeneration during homeostasis and disease (89–91). With the development of high efficiency genome editing tools, one group generated a novel porcine cell reporter model *via* CRISPR-Cas9 insertion of fused histone 2B (H2B) to green fluorescent protein (GFP) under the *ACTB* locus (92). With successful ubiquitous nuclear expression of GFP demonstrated within these pigs, the same group went on to insert the H2B-GFP sequence under control of leucine-rich repeat-containing G protein-coupled receptor 5 (*LGR5)*, a known biomarker expressed by ISCs (LGR5-H2B-GFP) (56, 84). Histologic sections of colon demonstrated nuclear GFP in the crypt base cell populations in this novel model. However, to truly utilize this translational model and isolate ISCs for study of intestinal disease, further work is necessary to conclude that these GFP expressing cells are in fact LGR5^+^ ISCs. With this information, researchers would then have access to the first ever large animal ISC reporter, making way for improved translational studies of colorectal cancer development and intestinal barrier function and repair.

## Future Directions: Transgenic Porcine Models of Inflammatory Bowel Disease

Inflammatory Bowel Disease (IBD) is a multifactorial disease that is typically categorized as Crohn's Disease (CD) or Ulcerative Colitis (UC). These syndromes are characterized by inflammation of the intestinal mucosa, influx of immune cells, and dysregulated cytokine production. Subsequently, patients suffer from episodes of abdominal pain, diarrhea, bloody stools, and weight loss (93). Historically, gene edited rodent models have been used to determine underlying etiologies and test therapeutic targets (94). Murine models of IBD include knock-outs of cytokines such as IL-10, TGF-β, IL-2, and IL-23. Loss of these cytokines disrupts regulation of inflammation in the intestine, leading to intestinal lesions similar to those seen in IBD (95–98). Mice have also been engineered to over express signals such as IL-7 or STAT4 to upregulate immune cell activity and induce IBD (99, 100). However, given the immunological differences known between man and mouse (5, 7), alternative models that better emulate human immune physiology are needed to test surgical interventions and pharmaceutical therapeutics. A host of factors have been identified to contribute to IBD including gut microbiota, environmental factors, and abnormal innate and adaptive immune responses (93). Pigs, with similar intestinal microbiota, immunology, and anatomy to humans, are the clear choice for IBD models (3, 6, 13, 18–23). Chemical-induction of IBD by dextran sulfate sodium (DSS) or trinitrobenzenesulfonic acid (TNBS) has been shown to reproduce intestinal lesions in the pig similar to those found in UC and CD, respectively (101–105). These models have been used to test advanced endoscopic techniques to correct strictures and supplemental amino acid therapy. To study IBD on a more mechanistic level within a translationally relevant large animal model, transgenic induced IBD porcine models, parallel to the IBD murine models, are needed to mimic the specific immune cell dysregulations seen with IBD.

## Conclusions

The need for large animal models, particularly porcine, to improve pre-clinical intestinal disease translational research is well known. As described in this review, innovative applications of gene-edited porcine models of Cystic Fibrosis, colorectal cancer, and ischemia-reperfusion injury have progressed both the mechanistic understanding of disease pathophysiology as well as led to novel therapeutic treatment development. With continued improvement of gene-editing systems such as CRISPR/Cas, additional porcine models to track intestinal stem cells or simulate disorders such as Inflammatory Bowel Disease can be made available to further progress translational intestinal disease research.

## Author Contributions

CS drafted and edited the manuscript. LG edited and revised the manuscript. CS and LG approved the final version of the manuscript.

## Conflict of Interest

The authors declare that the research was conducted in the absence of any commercial or financial relationships that could be construed as a potential conflict of interest.

## Publisher's Note

All claims expressed in this article are solely those of the authors and do not necessarily represent those of their affiliated organizations, or those of the publisher, the editors and the reviewers. Any product that may be evaluated in this article, or claim that may be made by its manufacturer, is not guaranteed or endorsed by the publisher.
